# Genetic Aspects of Preeclampsia and the HELLP Syndrome

**DOI:** 10.1155/2014/910751

**Published:** 2014-06-02

**Authors:** Kjell Haram, Jan Helge Mortensen, Bálint Nagy

**Affiliations:** ^1^Department of Obstetrics and Gynecology, Haukeland University Hospital, Bergen 5006, Norway; ^2^Department of Global Public Health and Primary Care, University of Bergen, Norway; ^3^1st Department of Obstetrics and Gynecology, Semmelweis University, Budapest 1088, Hungary

## Abstract

Both preeclampsia and the HELLP syndrome have their origin in the placenta. The aim of this study is to review genetic factors involved in development of preeclampsia and the HELLP syndrome using literature search in PubMed. A familial cohort links chromosomes 2q, 5q, and 13q to preeclampsia. The chromosome 12q is coupled with the HELLP syndrome. The *STOX1* gene, the *ERAP1* and 2 genes, the syncytin envelope gene, and the *−670 Fas* receptor polymorphisms are involved in the development of preeclampsia. The *ACVR2A* gene on chromosome 2q22 is also implicated. The toll-like receptor-4 (*TLR-4*) and factor V Leiden mutation participate both in development of preeclampsia and the HELLP syndrome. Carriers of the TT and the CC genotype of the *MTHFR C677T* polymorphism seem to have an increased risk of the HELLP syndrome. The placental levels of VEGF mRNA are reduced both in women with preeclampsia and in women with the HELLP syndrome. The BclI polymorphism is engaged in development of the HELLP syndrome but not in development of severe preeclampsia. The *ACE I/D* polymorphism affects uteroplacental and umbilical artery blood flows in women with preeclampsia. In women with preeclampsia and the HELLP syndrome several genes in the placenta are deregulated. Preeclampsia and the HELLP syndrome are multiplex genetic diseases.

## 1. Introduction

Preeclampsia is a multisystemic disorder in pregnancy with* de novo* hypertension and proteinuria occurring after the 20th gestational week and is characterised by hypertension and proteinuria, with or without oedema. The condition is associated with a reduced plasma volume, hemoconcentration, and increased vascular resistance. One of the chief targets is the kidneys and the clinical picture is dominated by hypertension and proteinuria [1–3]. The clinical findings of preeclampsia can manifest as either a maternal syndrome (hypertension and proteinuria with or without other multisystem abnormalities) or fetal syndrome (fetal growth restriction, reduced amniotic fluid, and abnormal oxygenation) [3]. The condition may cause serious maternal and fetal complications [3].

Women with preeclampsia may develop the HELLP syndrome (haemolysis, elevated liver enzymes, and low platelet), which occurs in 0.5% to 0.9% of all pregnancies and in 10% to 20% of women with severe preeclampsia. The syndrome may be complete or incomplete. The majority of women with the HELLP syndrome have hypertension and proteinuria but the condition may also occur without these [4]. Typical clinical symptoms of the HELLP syndrome are right upper abdominal quadrant or epigastric pain, nausea, and vomiting. The upper abdominal pain may be fluctuating, colic-like. Up to 30–60% has headache and about 20% visual symptoms. In the postpartum period the HELLP syndrome usually develops within the first 48 hours in women who have had proteinuria and hypertension prior to delivery. Excessive weight gain and generalized edema precede the syndrome in more than 50% of the cases [4]. However, it is important to recognize that women with a HELLP syndrome also may have unspecific symptoms, subtle signs, or viral syndrome-like symptoms which should not be overlooked. Many women report a history of malaise some days before presentation. The HELLP syndrome may cause different maternal, fetal, and neonatal complications which may be serious [4].

Preeclampsia and the HELLP syndrome present as symptomless placental conditions in the first hand and then as a maternal syndrome with lesions in different organs and various clinical symptoms [5].

Preeclampsia may occur without a fetus in the case of hydatidiform mole [6]. It is established that preeclampsia originates in the placenta [3, 7, 8]. It is conceivable that the HELLP syndrome also is a placenta-dependent condition [9, 10]. Following delivery of the placenta the maternal symptoms and signs of preeclampsia often disappear but a protracted course of a severe HELLP syndrome is not unusual [11].

A variety of molecular factors are involved in the pathophysiology of preeclampsia and the HELLP syndrome. The aim of this publication is to present a review and a comprehensive description of the genetic factors involved and to suggest modes of action in development of preeclampsia and the HELLP syndrome.

## 2. Search Strategy

An unsystematic search in PubMed was conducted by using the search words: “preeclampsia” or “HELLP” combined with “etiology,” or “gene” or combined with risk factors, respectively (2000–2013). Abstracts were read and the publication cited if it was considered adequate.

## 3. Risk Factors for Preeclampsia and the HELLP Syndrome

Several risk factors have been identified with increased risk of preeclampsia. Preeclampsia is generally regarded as a disease of the first pregnancy. The risk factors include chronic hypertension, renal diseases, obesity and insulin resistance, diabetes mellitus, preexisting thrombophilia, family history of preeclampsia, and smoking [3]. High body mass index (BMI) and metabolic syndrome 6 months postpartum were associated with preeclampsia but hardly with HELLP [12].

Most white women with HELLP are multiparous. Women with a HELLP syndrome are more likely than those in the control group to be >35 years old (33% compared with 22%), be nulliparous (67% compared with 43%), have had a previous gestational hypertensive disorder (9% compared with 7%), and have a multiple pregnancy (7% compared with 2%) [13].

The risk of preeclampsia increases in those who have limited sperm exposure with the same partner before conception. Most epidemiologic studies demonstrate that regular sexual intercourse of an extended period reduces the risk [14, 15]. Women with a history of abortion who conceived again with the same partner had nearly half the risk of preeclampsia compared to women without a history of abortion. In contrast, women with an abortion history who conceived with a new partner had the same risk as women without a history of abortion [16].

### 3.1. The “Root Cause”

Preeclampsia and the HELLP syndrome both originate in the placenta [17]. The “root cause” of preeclampsia is thought to be reduced placental perfusion [18]. It has been demonstrated that impaired extra villous trophoblasts (EVTs) invasion in the decidua and the spiral arteries and insufficient spiral artery remodeling may take place both in women with preeclampsia and in women with a HELLP syndrome [19]. Reduced placental perfusion interacts with preexisting maternal disorders, such as hypertension, renal disease, overweight, diabetes mellitus, obesity, lipid abnormalities, and insulin resistance. This will affect the maternal susceptibility to preeclampsia because it liberates bioactive factors which pass through the intervillous space into the maternal circulation inducing an inflammatory reaction and endothelial dysfunction [20–22].

HELLP and preeclampsia become clinically manifest during the second (early-onset form) or third trimester (late-onset form) of pregnancy but the initiating event occurs much earlier in gestation [23]. The resultant symptomatic second stage of preeclampsia, the maternal syndrome, usually develops after the 20th gestational week [8, 24]. The HELLP syndrome is also associated with widespread organ damage with different clinical signs in the second stage [4, 10].

However, implantation disorders can be detected in the first trimester, before 12 to 20 weeks' gestation, when the deep invasion of EVT and spiral artery remodeling occur and prior to failed vascular remodeling of the spiral arteries necessary to reduce placental perfusion [22, 25]. In some cases of preeclampsia and a HELLP syndrome aberrant restructuring of the uterine spiral arteries by invading trophoblasts between weeks 8 and 12 of gestation ultimately causes poor placental perfusion and placental ischemia [26]. Thus, factors which increase the risk of preeclampsia and are associated with abnormal implantation in early pregnancy might be the “real” “root cause” of preeclampsia [22, 25].

## 4. Genetic Considerations

### 4.1. Epidemiologic Studies

It has been recognised for many years that preeclampsia has different genetic components [27]. Both maternal and fetal genes appear to play etiological roles. Evidence for a role for paternally inherited fetal genomes comes from an analysis of data from the Norwegian Medical Birth Registry from 1967 to 1992, which identified almost 400,000 women who had had at least two pregnancies. It was possible from the records to distinguish between a pregnancy with the same parents and those where the mother or the father differed in the second pregnancy [28]. In the first pregnancies the risk of preeclampsia was above 3%, slightly increasing with mother's age. The risk was 1.7% among second pregnancies in mothers who had their second pregnancy with the same partner and 1.9% among second pregnancies in mothers who had changed their partner since the first pregnancy [28]. Men who were born from a preeclampsia pregnancy were at risk of fathering a preeclampsia pregnancy [29]. First degree female relatives of women with preeclampsia had a 5-fold higher risk and second degree relatives a 2-fold higher risk of developing preeclampsia than women without a family history of preeclampsia [30]. Studies of familial aggregation of preeclampsia indicate that genetic factors may contribute more than 50% of the variability in liability to preeclampsia [31, 32].

Women with a history of HELLP are at increased risk of preeclampsia (22–28%) and a HELLP syndrome (14–24%) in subsequent pregnancies, suggesting related pathogenic mechanisms [33, 34]. Sisters and children of a woman who has sustained a HELLP syndrome have increased risks of developing a HELLP syndrome in pregnancy [35].

## 5. Candidate Genes

In 1999 the results of a genome-wide scan of Icelandic families, which included 124 pedigrees of 343 women affected by preeclampsia, were published by Arngrímsson et al. The study revealed a maternal susceptibility locus for preeclampsia on chromosome 2p13 [36]. A genetic linkage to chromosomes 2q, 5q, and 13 q was demonstrated in an Australian/New Zealander familial preeclampsia cohort [37]. In a study by Lachmeijer et al. a genome-wide scan of Dutch affected sib-pair families uncovered a peak on chromosome 12q which was associated with the HELLP syndrome. It increased to a lod score of 2.1 in the HELLP families and almost disappeared in families with preeclampsia. This means that the HELLP syndrome and preeclampsia have a different genetic background [35].

Susceptibility genes at the 5q gene locus were also centered in a spectrum of families in a Norwegian preeclampsia cohort (1,139 cases and 2,269 controls) using (SNP) genotyping [37]. There was also evidence of a genetic association with preeclampsia for the endoplasmatic reticulum aminopeptidases 1 and 2 (*ERAP1 and 2*) genes. The* ERAP1* and* ERAP2* genes encode enzymes that play roles in blood pressure regulation via involvement of the renin-angiotensin system in addition to the innate immune system [37]. The* ERAP1* gene cleaves the cell surface receptors for the proinflammatory cytokine interleukin-1 (IL-1), IL-6, and the tumor necrosis factor *α* (TNF-*α*) thereby downregulating their signaling. The* ERAP1* gene can therefore have proinflammatory effects [37].

An association between the susceptibility activin receptor type 2 gene (*ACVR2A*) on chromosome 2q22 and preeclampsia (*n* = 1139) was demonstrated in the Norwegian cohort. DNA samples from 1139 cases (women with one or more preeclamptic pregnancies) and 2269 controls (women with normal pregnancies) were genotyped using the Applied Biosystems SNPlex high-throughput genotyping assay [38]. The fact that populations with different ancestors (Iceland/Norway-Australia/New Zealand) demonstrate a common maternal preeclampsia susceptibility locus on chromosome 2q22 may suggest a general role of this locus in the pathophysiology of preeclampsia. The* ACVR2A* gene may affect the activin A activity which is involved in EVT invasion in the decidua and the spiral arteries as well as in remodeling of the spiral arteries [38].

Goddard et al. conducted a large scale study evaluating 775 single nucleotide polymorphisms (SNPs) in 190 genes in women with preeclampsia (*n* = 294) and their offspring (*n* = 324) [39]. SNP discovery was performed by DNA sequencing, and genotyping was carried out in a high-throughput facility using the MassARRAY TM System. The most significant findings for an association with preeclampsia were the collagen, type I, alpha 1 (*COL1A1*) and* IL1A* genes for the maternal genotype and the plasminogen activator, urokinase receptor gene (PLAUR gene) for the offspring genotype. Notably, common candidate genes for preeclampsia, including methylene-tetrahydrofolate reductase (MTHFR) and nitric oxide synthase 3 (NOS3), were not significantly associated with preeclampsia [39].

Buimer et al. performed a study of the gene expression in placental tissues from normotensive pregnant women and women with preeclampsia and a HELLP syndrome. Firstly, comparison of Serial Analysis of Gene Expression profiles of 28 weeks' control placenta (available after idiopathic premature delivery) to a HELLP/preeclampsia placenta matched for gestational age identified 404 differentially expressed transcripts. Secondly, using sequential PCR, the expression levels of 37 of these transcripts in the placentas of healthy pregnant women (*n* = 36) and in the placentas of women with preeclampsia and a HELLP syndrome (*n* = 22) were analysed. Thirdly, nearest centroid classification determined the HELLP specific molecular signature consisting of the upregulated expression of genes encoding the vascular endothelial growth factor receptor (*FLT1*), leptin (*LEP)*, pappalysin 2 (*PAPPA2*), and WW domain containing transcription regulator 1 (*WWTR1*) combined with downregulated expression of the genes encoding cadherin-associated protein (*CTNNAL*), glutathione S-transferase pi (*GSTP1*), and calgranulin A (*S100A8*). This set discriminates the HELLP placenta from the placenta of healthy women and the placenta of women with preeclampsia with a 24% misclassification rate, independent of known risk factors like parity and ethnicity [23].

## 6. Some Selected Genes and Occurrence of Preeclampsia and HELLP

In the following the influences of different genes on development of preeclampsia and the HELLP syndrome according to various publications are presented. Genome-wide association studies (GWAS) have disclosed susceptibility genes for preeclampsia. The results seem compatible with the assumptions that unfavourable gene variants and interactions between genes regulating maternal-fetal interactions are involved in development of preeclampsia.  Table 1 summarizes selected gene effects on preeclampsia and the HELLP syndrome obtained after unsystematic search in PubMed.

## 7. The* STOX1* Gene

The 10q22 chromosomal region with genomic linkage to preeclampsia in Dutch females shows a parent-of-origin effect with maternal transmission of* Y153H* susceptibility allele of the* STOX1* gene [55]. The* STOX1 *gene, which is placentally expressed, was identified as a candidate gene for preeclampsia (*n* = 157) in a Dutch population [56]. Two studies (2007) in both a Dutch and a Finnish population did not support or confirm that the* STOX1* gene was involved in development of preeclampsia [57, 58]. However, the roles of* STOX1* on trophoblasts dysfunction have been confirmed in several studies. The* STOX1* gene is a key player in trophoblast dysfunction underlying early-onset preeclampsia [56, 59–61]. There does not seem to be any study which focuses on the* STOX1* gene and development of the HELLP syndrome.

## 8. The Syncytin Envelope Gene

The creation of the syncytium layer by cytotrophoblasts is an important step in the placentation process. Syncytin envelope gene is an essential gene mediating the cytotrophoblast cell-cell fusion and differentiation [40, 62, 63]. Decreased syncytin expression in cytotrophoblasts impairs the syncytiotrophoblast formation [64]. A study by Knerr et al. included women with preeclampsia (*n* = 16), women with a HELLP syndrome (*n* = 6), and healthy pregnant women (*n* = 30). After delivery, mRNA of syncytin, glyceraldehyde-3-phosphate dehydrogenase, and *β*-actin were analyzed in placental villi with use of quantitative real-time PCR. The syncytin mRNA levels were significantly lower in the chorionic villi in women with preterm preeclampsia and in women with the HELLP syndrome than in women with healthy pregnancies. A reduced placental expression of syncytin may contribute to altered cell-cell fusion processes in the placentogenesis and to a disturbed placental function [65].

The placenta was analyzed in a study by Langbein et al. comprising women with preeclampsia and IUGR (*n* = 8) and women with the HELLP syndrome and IUGR combined (*n* = 8). Total RNA was extracted from 50 to 100 mg of frozen placental tissues by mincing using a Mikro-Dismembranator (Braun Biotech, Sartorius AG, Goettingen, Germany) and purified using Trizol1 reagent (Invitrogen, Karlsruhe, Germany). The syncytin envelope placental gene expression in women with preeclampsia combined with IUGR was 5.4-fold lower and in women with the HELLP syndrome associated with IUGR was 10.6-fold lower along with a 1.8- and 1.9-fold significant increase in the apoptosis rate in the cytotrophoblasts compared with control subjects, respectively. This higher rate of apoptosis may be contributing to an inflammatory response [66].

## 9. Mannose-Binding Lectin (MBL) Gene Polymorphism

Insufficient invasion of the spiral arteries by trophoblast cells is associated with the etiology of preeclampsia and the HELLP syndrome. Mannose-binding lectin (MBL) is a component of the innate immune system. The* MBL gene* is located on chromosome 10 and is polymorphic at codons 52, 54, and 57. MBL is present in the spiral arteries, particularly in those containing endovascular trophoblasts. MBL-mediated activation of the complement cascade is an important event in destruction of invading EVTs [67].

Carriers of the MBL-54 B variant have been reported in 22–28% of European and North American populations [67]. Excessive MBL-mediated trophoblast damage would increase the possibility of insufficient EVT invasion of the spiral arteries. The subsequent hypoxia could initiate a sequence of events culminating in preeclampsia [67].

A study by Sziller et al. comprised women with preeclampsia (*n* = 51), women with the HELLP syndrome (*n* = 81), and healthy pregnant women (*n* = 184). DNA was extracted from buccal swabs of women with preeclampsia, women with HELLP syndrome, and healthy pregnant controls. Aliquots were tested for a single nucleotide MBL gene polymorphism at codon 54 by PCR and endonuclease digestion. There was significantly higher number of the TT genotype (25%) in women with the HELLP syndrome than in women with severe preeclampsia (9%) and in healthy (8%) pregnant women. It was suggested that maternal heterozygosity at codon 54 of the* MBL B allele* was protective against development of preeclampsia and HELLP syndrome. Homozygosity for the wild type allele A of the MBL-54 gene was more frequent among women with preeclampsia and the HELLP syndrome. Carriage of the* variant B allele* was also protective against development of IUGR in women with both preeclampsia and HELLP syndrome [67].

## 10. Factor V Leiden Mutation

Thrombosis and atherosis of the spiral arteries are often found in the placenta of women with preeclampsia, the HELLP syndrome, and IUGR [41, 42]. Women with a HELLP syndrome also had frequent acute atherosis in spiral arteries invaded by EVTs and in noninvaded spiral arteries (Robert Pijnenborg, personal communication). Factor V Leiden mutation is the cause of resistance to activated protein C which may lead to intravascular coagulation.

Lin and August performed a meta-analysis on genetic thrombophilias and preeclampsia assessing the relationship between the factor V Leiden* (1691 G-A)* SNP, the methylene tetrahydrofolate reductase* (MTHFR) 677 C-T* SNP, and the prothrombin* 20210 G-A* SNP in all case-control studies with data on these polymorphisms [68]. All investigations used PCR reaction and restriction fragment length polymorphism analysis of DNA obtained from blood or buccal cells in the case-control studies. The pooled odds ratio (OR) for the association of factor V Leiden mutation and all women with preeclampsia was 1.81 (95%) confidence interval (95% CI 1.14–2.87) and 2.24 (95% CI 1.28–3.94) for women with severe preeclampsia [68].

A publication from 2008 revealed a clear relationship between factor V Leiden mutation and women with a HELLP syndrome. Genotyping of the thrombophilic mutations was performed using the LightCycler technology. Maternal heterozygosity for factor V Leiden mutation was significantly more prevalent in women with a HELLP syndrome (*n* = 71) than in healthy control women (*n* = 79) (OR 4.45) (Table 2) [69]. No significant association was observed for maternal prothrombin mutation or MTHFR polymorphism. The study confirmed that women heterozygous for factor V Leiden have an increased risk of developing a HELLP syndrome, while the most frequent mutations of the prothrombin and MTHFR gene did not play a major role in the pathogenesis [69].

## 11. MTHFR Polymorphism, Homocysteine, Preeclampsia, and HELLP

It has been shown that alterations in the methionine homocysteine metabolism may be related to the systemic damage that leads to the classical clinical picture of preeclampsia [74]. A recent study evaluates the role of key enzymes in the methionine-homocysteine metabolism (MHM) in the physiopathology of preeclampsia. Plasma and placenta from pregnant women (32 controls and 16 preeclamptic women) were analysed after informed consent. Protein was quantified by western blot. RNA was obtained with RNA purification kit and was quantified by reverse transcriptase followed by real-time PCR. Identification of the* C677T* and* A1298C *methylene tetrahydrofolate reductase (*MTHFR*) SNPs and* A2756G* methionine synthase (MTR) SNP was performed using PCR followed by a high-resolution melting analysis. It was shown that women who develop preeclampsia are clinically distinguishable at 11–14 weeks of gestation. Key enzyme RNA expression is increased in preeclamptic women but this change is not reflected at the protein content. These results highlight a potential role of the MHM as a compensation mechanism in the presence of low levels of 2-methoxyestradiol (2-ME) [74].

The MTHFR gene plays a role in the metabolism of homocysteine. Nagy et al. conducted experiments on isolated DNA samples from the blood of women with severe preeclampsia (*n* = 101), women with the HELLP syndrome (*n* = 63), and healthy pregnant women (*n* = 73). The* MTHFR C677T* polymorphism was determined by quantitative real-time PCR. The mutant T allele was found in 45% of women with the HELLP syndrome, in 32% of the healthy pregnant women, and in 30% of women with severe preeclampsia [43]. The results showed no difference in the distribution of the MTHFR C677T genotypes between the healthy controls and women with preeclampsia. In the study, however, there was a substantial difference between the incidences of* MTHFR 677T* genotype among HELLP syndrome women and the other two study groups. Thus, the* MTHFR C677T* polymorphism might be involved in development of the HELLP syndrome [43].

## 12. Factor V Leyden Mutation,* G0210A* Mutation in the Factor II (Prothrombin) Gene, and MTHFR in Women with Thrombophilia

The mutation of the prothrombin gene is associated with an increased risk of both venous and arterial thromboembolism. Severe preeclampsia, the HELLP syndrome, and IUGR are associated with intervillous and/or spiral artery thrombosis and inadequate placental perfusion. A congenital thrombophilia disorder is the* G0210A* mutation in the factor II (prothrombin) gene which may lead to higher prothrombin production and to an increased risk of thrombosis [75].

A case-control study was performed by Benedetto et al. comprising women with preeclampsia (*n* = 111) and women with normal pregnancies (*n* = 111) matched for age and parity without previous thromboembolic disorders [45]. The women were tested for the A1691G mutation in the factor V Leyden mutation and the* G20210A* mutation of the factor II (progesterone) genes was amplified by PCR with Perkin Elmer Gene Amp 2400 (New Jersey, USA). Factor V Leiden mutation was found in a few women with preeclampsia and in healthy controls. Factor II* G20210A* mutation was also detected in some women with preeclampsia [45]. In the subgroup of women with the HELLP syndrome (*n* = 32), factor V Leiden mutation was detected in 3 women (9.3%) and factor II* G20210A* in 2 (6.2%). Thus, the prevalence of factor V Leyden mutation OR 2.29 (0.56–9.32) and factor II mutations OR 6.03 (0.65–47.54) was increased in women with preeclampsia. The thrombophilia mutations may interact with other pathogenic factors to determine the clinical features of the diseases and its complications [45].

In another study by Kupferminc et al. women were tested several days after delivery for the mutation of adenine to guanine at nucleotide 506 in the factor V gene (factor V Leiden), the mutation of cytosine to thymine at nucleotide 677 in the gene encoding* MTHFR*, and the mutation of guanine to adenine at nucleotide 20210 in the prothrombin gene the prothrombin gene* G20210A* mutation [76]. The* G20210A* mutation was found to occur more often in women with complications such as preeclampsia (*n* = 11) than in women without complications (*n* = 3) (10% versus 3%, resp.). Overall, 57 of the 110 women with obstetrical complications (52 percent) had at least one of the three thrombophilic mutations, as compared with 19 of the 110 women with normal pregnancies (17 percent) (OR 5.2; 95% 2.8 to 9.6) [76].

## 13. Vascular Endothelial Growth Factor (VEGF), Preeclampsia, and HELLP 

VEGF is produced by cytotrophoblasts and villous syncytiotrophoblasts in the placenta [46]. VEGF is an endothelial cell-specific growth factor and a regulator of physiological and pathological angiogenesis [47]. Nagy et al. performed a study to determinate three VEGF SNPs. The allele and genotype frequencies of* VEGF C −460T* SNP were determined in the maternal blood. DNA was isolated by using a silica adsorption method. The SNPs were determined by quantitative real-time PCR and melting curve analysis using LightCycler. Both carriers of the −*460TT* and the +*405CC* genotype of VEGF receptor genotype seemed to have an increased risk of development of a HELLP syndrome (*n* = 71) compared to healthy controls (*n* = 93). There were significant differences in the allele and genotype frequencies of* VEGF C *−*460T* SNP between the two study groups. The T allele was present in 71% in the HELLP group and in 54% of the healthy controls. The TT genotype occurred significantly more frequently in the HELLP group than in the control group (45% versus 22%). The TT genotype carriers had an increased risk of HELLP syndrome, which was independent of maternal age and primiparity (adjusted OR 3.95) (Table 2). Although the VEGF G +405C allele and genotype distributions did not differ significantly between the two groups, the CC genotype carriers were also found to have an increased risk of HELLP syndrome after adjustment for maternal age and primiparity (adjusted OR 3.67) (Table 2). The* VEGF C *−*2578A* SNP was not associated with the HELLP syndrome. The findings suggest that the VEGF polymorphisms, interacting with other genetic and environmental factors, could play a role in the development of the HELLP syndrome [77].

Sgambati et al. performed a study to determine the expression of VEGF in the placental tissue from pregnancies complicated by hypertension disorders of different clinical severity [72]. Placentas from women with gestational hypertension (*n* = 20), preeclampsia (*n* = 20), and preeclampsia with HELLP syndrome (*n* = 20) and from normotensive women (*n* = 20) were analyzed as a control group (gestational age comprised between 35 and 38 weeks). An immunohistochemical technique and a quantitative real-time PCR analysis to measure mRNA levels were employed. The levels of VEGF mRNA were higher in women with gestational hypertension and lower in women with preeclampsia with HELLP syndrome compared to the levels of VEGF mRNA in control women [72]. The different expression of VEGF in the placenta of the pathological cases is probably related to hemodynamic changes that take place in these disorders, in order to attempt restoration of a normal uteroplacental flow [48].

## 14. The Angiotensin-Converting Enzyme (ACE) I/D Polymorphism

Local prostacyclin stimulates the uteroplacental renin-angiotensin system (RAS) and induces release of angiotensin-II (Ang-II) from trophoblasts. This improves the uteroplacental blood flow by increasing perfusion pressure and by forming an extra stimulus of prostacyclin and nitric oxide (NO) release from uteroplacental vessels [48, 78].

The renin-angiotensin system is one of the mediators of the EVT invasion and remodeling of the spiral arteries. In a study women with preeclampsia (*n* = 106) were genotyped for the* ACE I/D *polymorphism. Genomic DNA was extracted from leukocytes using a QIAmp Blood Kit (QIAGEN, Hilden, Germany) [79]. The* ACE I/D* polymorphism seemed to affect the uteroplacental and umbilical artery (UA) blood flows and the recurrence of preeclampsia. The UA resistance index was significantly lower in the II genotype, higher in DD, and intermediate in I/D genotype carriers. The UA pulsatility index values were significantly higher in the DD group than in the I/D and II genotype [79]. It was concluded that in women with a history of preeclampsia the* ACE I/D* polymorphism affects both uteroplacental and umbilical blood flows and the recurrence of preeclampsia [79].

## 15. The BclI Polymorphism of the Glucocorticoid Receptor (GR) Gene

The glucocorticoid receptor (GR) gene is the receptor to which cortisol and other glucocorticoids bind. The GR gene is expressed in almost every cell in the body and regulates genes controlling the development, metabolism, and immune response. A study by Bertalan et al. comprised healthy pregnant women (*n* = 300), women with severe preeclampsia (*n* = 150), and women with a HELLP syndrome (*n* = 17) [80]. Total genomic DNA was isolated from peripheral blood lymphocytes using a QIAamp DNA Blood Mini Kit (Qiagen GmbH, Hilden, Germany) and the blood spot samples were denatured by heat inactivation. The BclI and the N363S polymorphisms of the GR gene were determined by allele-specific PCR. There were no significant differences in carrier and allelic frequencies of the* N363S* and* ER22/23EK* polymorphisms between healthy pregnant women and those with severe preeclampsia. However, the allelic and carrier frequencies of the BclI polymorphism were significantly higher in women with HELLP syndrome compared to healthy pregnant women (OR 2.89) and compared to those with severe preeclampsia (OR, 2.56) (Table 2). The observations suggest that, among pregnant women, the BclI polymorphism is associated with development of the HELLP syndrome but not of severe preeclampsia. Interestingly, the BclI carrier status had a significant impact on clinical laboratory parameters of women with the HELLP syndrome. The aspartate aminotransferase (AST), lactate dehydrogenase (LDH), and alkaline protease (ALP) levels were significantly higher whereas the platelet count tended to be lower in BclI carriers than in noncarriers [80].

## 16. Polymorphism of Microsomal Epoxide Hydrolase Gene (*EPHX*)

Genetic polymorphisms have been described in the human gene encoding for microsomal* EPHX*. Two of these polymorphisms, 113Tyr→His in exon 3 and 139His→Arg in exon 4, are associated with either a decreased or an increased enzyme activity, respectively [49]. A study by Zusterzeel et al. from 2001 focused on whether or not the genetic variability in the* EPHX *gene could contribute to preeclampsia and the HELLP syndrome Genomic DNA was isolated from whole blood using the WizardTM genomic DNA purification kit (Promega, Madison, WI, USA). A more frequent high activity genotype Tyr113→Tyr113 in exon 3 (29%) was found in women with preeclampsia than in control subjects (16%) (OR 2.0) (Table 2). In women with a history of preeclampsia no difference in epoxide hydrolase genotypes was found between women who either did or did not develop the HELLP syndrome [49]. Women with the high activity genotype in exon 3, which could reflect differences in metabolic activation of endogenous or exogenous toxic compounds, may have enhanced susceptibility to preeclampsia [49].

## 17. Polymorphism of the Fas Receptor

Fas and Fas Ligand (FasL) are classical transmembrane proteins that belong to the tumour necrosis factor receptor super family (TNFRSF). FasL is localized to both syncytiotrophoblasts and cytotrophoblasts in the placental chorionic villi [50]. FasL and its receptor, Fas, are known to play an important role in regulation of the immune response. Fas is highly expressed on activated T cells, B cells, NK cells, and macrophages [50]. Sziller et al. reported that a single adenine (A) to guanine (G) polymorphism at position −670 in the Fas receptor (*TNFRSF6)* results in decreased Fas synthesis. A study by Sziller et al. comprised women with a HELLP syndrome (*n* = 84) and normotensive women (*n* = 83) [51]. Cells from the buccal mucosa were obtained by rotating cotton swab within 1 hour after delivery. Genotype and allele frequencies were determined by direct counting and then dividing by number of chromosomes to obtain allele frequency and by the number of women to obtain genotype frequencies [51]. It was found that homozygous carriers with the −670 AG or GG genotype were more likely to develop the HELLP syndrome than those homozygous for the wild type of the Fas receptor (TNFRSF6−670A/A) (OR 2.7) (Table 2) [51]. The TNFRSF6−670 polymorphism is linked to decreased apoptotic potential of maternal T cells due to polymorphism in the Fas receptor at −670 which is thought to contribute to a prolonged capacity of maternal lymphocytes to recognize and destroy EVTs during invasion of the decidua and the spiral arteries which may lead to development of preeclampsia or HELLP syndrome [51].

## 18. Maternal* TLR-4* and* NOD2* Gene

Toll-like receptors (TLR) are considered to be the most important class of pattern-recognition receptors (PRRs) involved in host defence against a variety of microbes. TLRs are central components of the innate immune system [81]. Toll-like receptors (TLRs) are central components of the innate immune system [81]. The allelic variants of the innate immune system, the toll-like receptor-4 (*TLR-4*) and the* NOD2* gene (an apoptosis regulator) variants, were investigated by van Rijn et al. 6 months after delivery in primiparous women with a history of an early-onset preeclampsia (*n* = 340) of whom 177 developed the HELLP syndrome; 113 women with an uneventful pregnancy were controls [52]. Genomic DNA was isolated from blood, using standard commercially available kits (Gentra Systems, Minneapolis, MN, USA). Two common missense mutations of the* TLR-4* gene (GenBank: NM_138554; OMIM: 603030) were detected by PCR. The highest frequency of the TLR-4 was observed in women who developed the HELLP syndrome (adjusted OR 4.1) (Table 2). Combined positivity for any of the* TLR-4* and* NOD2* allelic variants and high levels of IL-6 were 6.9-fold more common in women with a history of early-onset preeclampsia than in healthy pregnant women (OR 6.9) (Table 2) [52]. In women with preeclampsia a persistent TLR-4 signal could reverse the CD4^+^CD25^bright^ Treg cell-mediated immunosuppression [82]. This means that the TLR-4 pathway and the innate immune system might be involved in development of both the HELLP syndrome and early-onset preeclampsia [52].

## 19. Leptin Gene (*LEPR*)

During pregnancy hormonal readjustment leads to increased leptin transcription. Women with preeclampsia display significantly higher serum leptin levels than healthy pregnant women. High leptin levels correlated with the blood pressure values [53]. Chronic hyperlipemia was associated with an increased release of the vasoconstrictor endothelin as well as an activation of the sympatric nerve system which may cause vasoconstriction [83].

The leptin receptor (*LEPR*) is a member of the super family of cytokine receptors. The LEPR gene is transcribed in the villous and EVTs. Its serum level correlates with the leptin concentration in peripheral blood [84]. The leptin gene polymorphisms were examined in women with preeclampsia (*n* = 40) and in control women (*n* = 29) by Muy-Rivera et al. [73]. Maternal plasma leptin and SLR concentrations were measured using enzyme immunoassays (Diagnostic Systems Laboratory, Inc., Webster, Texas, USA). Elevated leptin concentrations (14.5 ng/mL) were associated with a 3.8-fold increased risk of preeclampsia (OR 3.8) (Table 2) whereas low soluble leptin receptor (*SLR*) (<28.5 ng/mL) was associated with a 6.3-fold increased risk of preeclampsia 6.3-fold (OR 6.3, 95%CI 1.7–23.2). On the other hand, the I/II genotype was associated with a 3.8-fold increased risk of preeclampsia (OR 3.8) (Table 2) [73].

The frequency of* LEPR 223AA* genotype in women with severe preeclampsia (*n* = 24) and healthy pregnant women (*n* = 107) was analysed by Rigó Jr. et al. Peripheral blood samples taken for routine laboratory investigations were used for genotyping the two common DNA sequence variants in exons 4 and 6 of the LEPR gene using the PCR restriction fragment length polymorphism (*RFLP*). Genomic DNA was extracted using the standard phenol-chloroform extraction procedure [54]. Women with the* LEPR 223G* allele (*223A/G* or* 223G/G* genotype) had almost a doubled risk of developing severe preeclampsia compared with women with the 223A/A genotype (adjusted OR 1.92) (Table 2) [54]. Thus, the LEPR gene is associated with increased risk of developing preeclampsia.

## 20. MicroRNA (MiRNA)

MiRNAs are noncoding 20–30 bp nucleotide RNAs with negative regulation functions which control the gene expression by binding to messenger RNAs and play roles in biological functions like cell proliferation, differentiation, apoptosis, cardiovascular disease, and carcinogenesis [85, 86]. The MiRNAs are abundantly expressed in the placenta. Zhu et al. found several microRNA clusters on chromosomes 19q13.42, 13q31.3, Xq26.2, Xq26.3, and 14q32.31 (a human imprinted region) which were expressed differentially in the placenta of women with preeclampsia. The results showed that in women with preeclampsia microRNAs were deregulated, suggesting involvement of microRNAs in the pathogenesis [87].

## 21. Chorionic Villous Samples (CVS) and Deregulation of Placental Genes

The human placenta is prerequisite for the development of preeclampsia and the HELLP syndrome. Several studies have focused on placental genes. Farina et al. analysed chorionic villous samples (CVS) from the 11th gestational week from women who developed preeclampsia (*n* = 10) and healthy controls (*n* = 50) with microarray. Altered expression was found among several genes including those involved in invasion of EVT (*Titin*), in inflammatory stress (*Lactotransferrin*), endothelial aberration (*Claudin 6*), angiogenesis (*Vasohibin 1*), and blood pressure control (*Adducin 1*). CVS showed an aberrant gene profile prior to development of preeclampsia [88]. Founds et al. performed 160 CVS at 10–12-week gestation. Thirty-six differentially expressed genes were identified in the preeclampsia placenta. Decidual gene deregulation was prominent [89].

## 22. Microarray Study of Women with Early-Onset Preeclampsia and the HELLP Syndrome

Várkonyi et al. conducted a microarray study of women with early-onset preeclampsia and women with the HELLP syndrome. Placental specimens were obtained by Cesarean sections from women with early-onset preeclampsia and women with the HELLP syndrome. Placental specimens were collected after birth from control women who delivered vaginally preterm or at term. After histopathological examination, fresh-frozen placental specimens were used for microarray profiling and validation by quantitative real-time PCR. Out of the 350 differentially expressed genes in women with preeclampsia and 554 genes in women with the HELLP syndrome, 224 genes (including* LEP, CGB, LHB, INHA, SIGLEC6, PAPPA2, TREM1*, and* FLT1*) changed in the same direction (elevated or reduced) in both syndromes. Enrichment analyses revealed similar biological processes, cellular compartments, and biological pathways enriched in early-onset preeclampsia and the HELLP syndrome. However, some processes and pathways (e.g., cytokine-cytokine receptor interaction) were overrepresented in women with the HELLP syndrome [90].

In accordance with the altered trophoblast pathophysiology in early-onset preeclampsia, many of the most differentially regulated genes in women with preeclampsia (*LEP*,* CGB*,* LHB*,* INHA*,* SIGLEC6*,* PAPPA2*, and* FLT1*) have predominant or unique expression in the syncytiotrophoblasts [91]. The results of the study by Várkonyi et al. may imply that trophoblastic metabolic signals overactivated the reproductive axis in women with early-onset preeclampsia and women with the HELLP syndrome. Moreover, enrichment analyses also revealed the overrepresentation of biological processes, molecular functions, and pathways that are related to uteroplacental vascular insufficiency and placental oxidative stress, known to play key roles in the pathophysiology of early-onset preeclampsia [8, 21, 92].

## 23. Discussion and Future Directions

The present review highlights several of the genetic factors playing roles in development of preeclampsia and the HELLP syndrome. A variety of genes are involved which also interact. The mechanisms are very complicated. Gene variants in the Fas receptor, the VEGF gene, and the coagulation factor V Leiden mutation are associated with increased risk of the HELLP syndrome compared to healthy women [51, 69, 77]. Variants in the Bell polymorphism [93] and the* TLR-4* increased the risk of HELLP significantly more than the risk of preeclampsia [52]. A weakness of our publication is that it is an unsystematic review carrying risk of missing important references. A weakness of many of the studies is the low number of cases which makes the genetic evaluation less certain. In addition, different criteria have been used in the diagnosis of preeclampsia and the HELLP syndrome. Criteria for incusing and exclusions also differ in evaluating case-control studies of thrombophilia [70].

Interestingly, overall gestational age at delivery was earlier in the studies in which higher ORs were reported which may imply a more severe preeclampsia. For example, the cases of preeclampsia reported by Scholz et al. were delivered at less than 28 weeks. In this group the factor V Leiden OR for preeclampsia was 5.7 [44]. In contrast, the cases reported by De Groot et al. which were delivered at 34-week gestation no association between preeclampsia and factor V Leiden mutation was observed [94].

Retrospective studies suggest that the heritable allelic variations, particularly the uteroplacental renin-angiotensin system with defective placental vascular development, could become the cornerstone for the genetics of preeclampsia [74].

In a study it was shown that decidual dendritic cells (DC) stained and reacted differently with decidual natural killer (dNK) cells in women with the HELLP syndrome compared to women with preeclampsia [44]. In another study, apoptosis, proliferation, and FasL expression were higher in villous trophoblast in women with the HELLP syndrome than in the women with preeclampsia and the control group. Placentas from a preeclampsia group had higher levels of apoptosis, lower FasL expression, and no difference in proliferation compared with the control group. The findings in this study suggested different pathophysiologic mechanisms in development of preeclampsia and the HELLP syndrome [95].

In the future there is a need of multicenter studies with a large number of cases using the latest technology, such as array comparative genomic hybridization and massive parallel sequencing, to reveal the genetic changes which have occurred on the whole genome in women with preeclampsia and the HELLP syndrome. The microarrays with a high number of SNPs also seem promising. The use of free nucleic acids is still at the early stage. More studies are needed also to apply DNA, RNA, and miRNA measurements. It is possible that a massive parallel sequencing might contribute to a better understanding of the pathophysiology of preeclampsia and the HELLP syndrome.

## Figures and Tables

**Table 1:** Gene types connected to preeclampsia and the HELLP syndrome-mode of actions.

Gene	Effect on preeclampsia or the HELLP syndrome	References
The STOX1 gene	A key player in trophoblast dysfunction underlying early-onset preeclampsia	[40]

Syncytin envelope gene	A reduced expression may disturb placental function and increase rate of apoptosis in cytotrophoblasts	[41]

MBL gene polymorphism	Excessive MBL-mediated trophoblast damage may cause insufficient EVT invasion of the spiral arteries. Maternal heterozygosity at codon 54 of the MBL B allele protects against preeclampsia and HELLP	[42]

Factor V Leiden mutation	Increase risk of preeclampsia and HELLP	[43, 44]

MTHFR C677T polymorphism	Involved in development of the HELLP syndrome.	[45]

G0210A mutation of factor II (prothrombin) gene	Involved in development of preeclampsia	[46, 47]

The VEGF TT-460 SNP genotype	Carriers had an increased risk of HELLP syndrome and could play a role in development of the HELLP syndrome	[48]

ACE I/I/D polymorphism	The renin-angiotensin system is a mediator of the EVT invasion and remodeling of the spiral arteries. The ACE I/D polymorphism seemed to affect the uteroplacental and umbilical artery (UA) blood flows and the recurrence of preeclampsia	[49]

BclI polymorphism of the GR gene	The BclI polymorphism is associated with development of HELLP syndrome but not of severe preeclampsia	[50]

Polymorphism of EPHX gene	High activity genotype in exon 3, which could reflect differences in metabolic activation of endogenous or exogenous toxic compounds, may have enhanced susceptibility to preeclampsia	[51]

NFRSF6-670 polymorphism	Homozygous carriers with the -670 AG or GG genotype are more likely to develop the HELLP syndrome than those homozygous for the wild type of the Fas receptor (TNFRSF6-670A/A)	[52]

TLFR-4 gene	TLR-4 pathway and the innate immune system might be involved in development of both early-onset preeclampsia and the HELLP syndrome	[53]

Leptin gene (LEPR)	The LEPR gene and its serum level correlate with the leptin concentration in peripheral blood. The LEPR gene is transcribed in the villous and EVTs	[54]

**Table 2:** OR impact on preeclampsia and HELLP.

Gene variant	Preeclampsia compared to	HELLP compared to	Number	OR (95% CI)	References
Factor V Leiden mutation		Healthy pregnancy	71	4.45 (1.31–15.31)	[69]
Factor Leiden mutation 169G-A SNP	Healthy pregnancy		1,798	1.81 (1.14–2.87)	[70]
Factor V Leiden mutation		Healthy pregnancy	32	2.29 (0.56–9.32)	[45]
Factor II prothrombin mutation		Healthy pregnancy	32	6.03 (0.65–47.54)	[45]
Three mutations		Healthy pregnancy	7,522	1.84 (1.14–2.87)	[71]
*MTHFR 677C-T* SNP	Healthy pregnancy		2,250	1.01 (0.79–1.29)	[1]
*VEGF C −460T* SNP		Healthy pregnancy	71	Adjusted OR 3.95 (1.51–6.08)	[72]
*VEGFG 405C *SNP		Healthy pregnancy	71	Adjusted OR 3.67 (1.05–1.75)	[72]
BclI polymorphism of GR gene		Healthy pregnancy	17	Adjusted OR 2.89 (1.45–5.74)	[49]
BclI polymorphism of GR gene	Severe preeclampsia		150	2.56 (1.45–5.74)	[49]
Polymorphism of EPHX gene	Healthy pregnancy		87	2.0 (1.2–3.7)	[49]
Fas *TNFRSF6 *gene		Healthy pregnancy	84	2.7 (1.2–5.9)	[51]
TLR-4 gene		Healthy pregnancy	177	4.1 (1.7–9.8)	[52]
Combined TLR-4 and NOD2 variant and high levels of IL-6	Healthy pregnancy		177	6.9 (2.1–23.2)	[52]
I/II genotype of LEPR gene	Healthy pregnancy		40	3.8 (0.8–18.0)	[73]
*LEPR* 223G allele	LEPR 23A/A genotype		24	1.92 (1.07–3.41)	[54]
